# An SNP-Based Genetic Map and QTL Mapping for Growth Traits in the Red-Spotted Grouper (*Epinephelus akaara*)

**DOI:** 10.3390/genes10100793

**Published:** 2019-10-12

**Authors:** Xiang Wang, Shoujia Jiang, Leyun Zheng, Ling Xiao, Xinhui Zhang, Dengdong Wang, Shuisheng Li, Qiong Shi, Shuiqing Wu, Haoran Lin, Xinxin You, Yong Zhang

**Affiliations:** 1 South China Sea Marine Survey and Technology Center, SOA, Guangzhou 510275, China; wangxiang@smst.gz.cn; 2 Laboratory for Marine Fisheries Science and Food Production Processes, Qingdao National Laboratory for Marine Science and Technology, Qingdao 266373, China; Lsslhr@mail.sysu.edu.cn; 3 Shenzhen Key Lab of Marine Genomics, Guangdong Provincial Key Lab of Molecular Breeding in Marine Economic Animals, BGI Academy of Marine Sciences, BGI Marine, BGI, Shenzhen 518083, China; jiangshoujia@genomics.cn (S.J.); zhangxinhui@genomics.cn (X.Z.); shiqiong@genomics.cn (Q.S.); 4 Fisheries Research Institute of Fujian, Xiamen 361012, China; lyzheng69@sina.com (L.Z.); wushuiqing90@163.com (S.W.); 5 State Key Laboratory of Biocontrol, Guangdong Provincial Key Laboratory for Aquatic Economic Animals and Guangdong Provincial Engineering Technology Research Center for Healthy Breeding of Important Economic Fish, School of Life Sciences, Sun Yat-Sen University, Guangzhou 510275, Chinawangdengdong@hotmail.com (D.W.); Lshuish@mail.sysu.edu.cn (S.L.); 6 Southern Marine Science and Engineering Guangdong Laboratory (ZhanJiang), Fisheries College, Guangdong Ocean University, Zhanjiang 524088, China

**Keywords:** *Epinephelus akaara*, genetic map, RAD, growth-related traits, QTL, network analysis

## Abstract

The red-spotted grouper (*Epinephelus akaara*) is one of the most commercially important aquatic species in China. However, its seedstock has low larval survival rates, and its stability is confronted with the danger of overexploitation. In this study, a high-density genetic map was constructed using 3435 single nucleotide polymorphisms (SNPs) from 142 first generation (F_1_) full-sib offspring and two parents of a red-spotted grouper population. The total genetic length of the map was 2300.12 cM with an average intermarker distance of 0.67 cM. Seventeen genome-wide significant quantitative trait loci (QTLs) for growth-related traits were detected on 24 linkage groups, including 5 QTLs for full length, 7 QTLs for body length, and 5 QTLs for body weight. The contribution values of explained phenotypic variance ranged from 10.7% to 12.9%. Moreover, 13 potential candidate genes for growth-related traits were identified. Collectively, these findings will be useful for conducting marker-assisted selection of the red-spotted grouper in future studies.

## 1. Introduction

The red-spotted grouper (*Epinephelus akaara*) is a popular, edible aquatic species in many Asian countries [1]. Like other grouper species, the red-spotted grouper is a protogynous hermaphrodite that is first differentiated as female and later changes to male at six years of age. Therefore, male red-spotted groupers are the limiting factor in the mass production of fingerlings and large-scale aquaculture of this commercial fish [2]. In order to avoid decreasing seedstock sources of the red-spotted grouper, many countries have strengthened the protection and management of this wild species’ resources. The development of DNA markers and new genetic methods may contribute to the construction of a high-density genetic map and the identification of quantitative trait loci (QTLs), and have contributed to the conservation of this species. Moreover, single nucleotide polymorphism (SNP) markers are commonly used to evaluate the genetic population structure due to their high polymorphism and codominant inheritance [3]. 

With the development of next-generation sequencing (NGS) technology, the construction of high-quality linkage maps for various economically important fish species has been facilitated. Several methods have been simultaneously developed for high-throughput marker discovery and genotyping using restriction enzymes, including restriction site-associated DNA sequencing (RAD-Seq) [4]. Thus, developing a high-quality genetic linkage map is a vital prerequisite for QTL mapping and increasing the efficiency of marker-assisted selection (MAS), thereby enhancing genetic progress. Such maps influence the relative positioning of diverse marker loci and help identify QTLs related to many traits. To date, researchers have constructed many linkage maps and developed QTLs of fish species including Atlantic salmon (*Salmo salar*) [5], Japanese flounder (*Paralichthys olivaceus*) [6], rainbow trout (*Oncorhynchus mykiss*) [7,8], Chinese mitten crab (*Eriocheir sinensis*) [9], southern catfish (*Silurus meridionalis*) [10], Pacific oyster (*Crassostrea gigas*) [11], snapper (*Chrysophrys auratus*) [12], and northern snakehead (*Channa argus*) [13]. The first high-density genetic linkage map for groupers (orange-spotted grouper, *Epinephelus coioides*) was constructed previously [14], which revealed that 27 significant QTLs and 17 genes were associated with growth-related traits [15]. Additionally, microsatellite-based linkage maps for the red-spotted grouper [16], white grouper (*Epinephelus aeneus*) [17], and kelp grouper (*Epinephelus bruneus*) have been published [18]. Recently, the genome assembly of red-spotted grouper was also reported [19]. However, an SNP-based genetic map and QTL mapping of the red-spotted grouper have not yet been reported.

Computational tools provide a promising alternative for helping identify biologically significant QTLs and SNPs. In this study, two open software packages were used to study the red-spotted grouper in RAD analysis data. STRING (a database of known and predicted protein-protein interactions) collects and integrates this information by consolidating known and predicted protein–protein association data for a large number of organisms [20]. Cytoscape software visualizes interaction networks and pathways [21]. These open-source software packages have also been used to study high-throughput expression data for other fish, including zebrafish (*Danio rerio*) [22], orange-spotted grouper [23], and rainbow trout [24].

In this study, we constructed a high-quality genetic linkage map and developed QTLs for growth-related traits in the red-spotted grouper. In total, 17 growth-related QTLs were identified, and 13 related genes were discovered. These findings will be beneficial for MAS with regard to growth traits in the red-spotted grouper.

## 2. Materials and Methods

### 2.1. Mapping Family and DNA Isolation

The red-spotted grouper first generation (F_1_) full-sib family was collected from a large seawater farm in Xiaodeng Island, Fujian Province, China, during July 2016. The fish were acclimatized for one month under controlled conditions. Approximately 15,000 progenies were stocked into a 600-m^2^ (30 m × 20 m × 1.5 m) pond at an initial density of 17 fish per m^3^. All fish were fed at 08:00 h and 16:00 h every day. During this period, the water quality parameters were monitored as follows: water temperature 27.5 ± 1.5 °C, salinity 29 ± 1‰, dissolved oxygen 10 ± 0.5 mg/L, pH 8.0 ± 0.2, and un-ionized ammonia 0.02 ± 0.1 mg/L. After 50 days of culturing, 144 individuals were randomly selected for phenotypic measurements, and the average weight of the fish was 4.2 g. Three growth-related traits, body weight (BW), full length (FL), and body length (BL), were recorded as phenotypic data (Table S1). 

Genomic DNA was extracted from the fresh muscle tissues of 144 individuals (142 offspring, two parents) following the phenol-chloroform protocol [6]. DNA quality was evaluated using a Qubit Fluorometer (Invitrogen, MA, USA) and electrophoresis on 0.6% agarose gel. All experiments were conducted following the regulations of the Animal Ethics Committee and were approved by the Institutional Review Board on Bioethics and Biosafety of BGI (No. FT14015).

### 2.2. Restriction Site-Associated DNA Sequencing

Ten RAD-Seq libraries were prepared for the 142 offspring and two parents following standard protocol [25]. Briefly, 1 µg of genomic DNA from each sample was digested with PstI restriction enzyme (Thermo Scientific, MA, USA) and incubated for 10 min in FastDigest buffer (total volume: 30 µL) at 65 °C. Barcode adapters (F: AAGTCGGAGGCCAAGCGGTCTTAGGAAGACAA, R: AAGTCGGATCGTAGCCATGTCGTT CTGTGAGCCAAGGAGTTG) containing a sample-specific nucleotide code were designed following the standard Illumina adapters design protocol. A total of 10 µmol of unique barcode adapters from each DNA sample was added to the reaction system. Sixteen samples were pooled within each tube. Nine pools were collected, and fragments between 300 and 500 bp were selected. Finally, sequencing was conducted using the Illumina Hiseq 4000 platform (Illumina, CA, USA) with 150-bp paired-end strategy. 

### 2.3. Sequence Data Analysis and Genotyping

All RAD sequence analyses and genotyping were conducted using the non-reference genomes software program Stacks v2.4 [26]. First, the data reads without a barcode or expected restriction enzyme motif were filtered; the filtering principles included (1) base quality ≥25; (2) depth between 5 and 300; and (3) at least one heterozygote from the parents. Second, the Stacks pipeline was used to build loci from short-read sequences using the following process: (1) RAD tags were clustered until exact matching by ustacks (-m 2 -M 2 -p 15); (2) cstacks (-b 1 -n 3 -p 15) was set to collect variations; and (3) retained reads were sorted into loci by sstacks (-p 15 -b 1). Lastly, clean tag reads of offspring were aligned to the parents’ SNP regions, where the genotypes of individuals were determined by the reference sample genotype. 

### 2.4. Genetic Map Construction

JoinMap v4.1 software was used to assign linkage groups (LGs) based on the cross population (CP) algorithm [27]. The genetic map was constructed using Lep-Map software [28]. All markers were filtered manually to remove obvious Mendelian errors from the offspring genotype data. The filtered SNPs could be used for further genetic map construction. First, the LG assignment was obtained using the JoinMap v4.1 software. Then, Lep-MAP was used to construct the genetic map using genotype data from the F_1_ red-spotted grouper population using the following process: (1) linkage analysis was performed for at least 80% of the markers; (2) the default value of data tolerance (*p*-value = 0.01) was used to filter out highly segregated markers (χ^2^ test, *p* < 0.01); (3) markers were assigned to LGs and executed with a logarithm of odds (LOD) score ranging from 2 to 10; and (4) markers within each LG were ordered. The error parameters were also calculated.

### 2.5. QTL Mapping Analysis and Gene Annotation

The MapQTL v6 software was used to perform the QTL analyses [29]. Composite interval mapping (CIM) was adopted with a walking speed of 1 cM [30]. The significance of each QTL interval was tested by a likelihood-ratio statistic. The LOD-supported intervals were constructed as 95% confidence intervals [31]. One thousand permutations were used, and the significance (*p* < 0.05) of the whole genome was determined based on the threshold level of LOD. The genes located in the QTL regions were supposedly growth-related genes, which were identified by QTL mapping. First, markers in the red-spotted grouper QTL regions were identified, and the corresponding marker sequences were selected from the reference parents based on their SNP ID. Second, sequences were mapped based on the position of the orange-spotted grouper genome scaffolds. Lastly, upstream and downstream coding gene functions were identified by a BLASTX (Basic Local Alignment Search Tool) search in the National Biotechnology Information Center. The sequencing reads of the orange-spotted grouper genome were deposited in the ENA Public Database (accession numbers: ERS2656083, SAMEA4836214; ERS2656082, SAMEA4836213).

### 2.6. Network Analysis Between the Candidate Genes

Genes in the connection groups were determined using the STRING online software by inputting SNP ID sequences [20]; the organism (*Danio rerio*) can be selected by clicking on the arrow or directly typing the name into the related input field. Two criteria were applied for detecting important nodes: high confidence with score = 0.15 and no more than 10 interactors. Then the network images were generated; clicking on a node provides details about the protein. Lastly, Cytoscape v3.7.1 software was used to visualize and analyze the molecular and interaction networks [21].

## 3. Results

### 3.1. Genotyping by Sequencing

In total, 114 Gb of raw data were generated from 10 RAD libraries, and 98 Gb of clean data were retained after data trimming by removing low-quality raw reads. Clean reads of separated individuals were stored in the CNSA (CNGB Nucleotide Sequence Archive) public database (CNP0000564). Then, loci in a set of individuals were identified, and the genotype of each locus was determined. Collectively, 20,798 SNPs were obtained, including four segregation types: <ef × eg> (n = 740), <hk × hk> (*n* = 2373), <lm × ll> (*n* = 7430), and <nn × np> (*n* = 10,255). 

### 3.2. Construction of the Genetic Map

**Figure 1:**
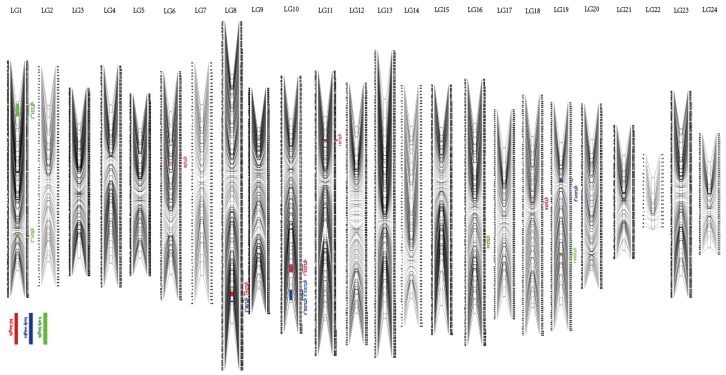
LG lengths and marker distributions of a high-resolution genetic map of the red-spotted grouper. Quantitative trait loci (QTL) names are shown on the left side of each LG. Red, green, and blue represent the full length, body length, and body weight traits, respectively.

A high-resolution genetic map of the red-spotted grouper was constructed using JoinMap and Lep-MAP for the first time. The successful mapping of 3435 SNPs in 24 LGs was consistent with the haploid chromosome number of the red-spotted grouper (2*n* = 48) [32]. The total genetic distance of the linkage map was 2300.12 cM with an average intermarker distance of 0.67 cM. The SNP number of each LG ranged from 20 to 251 (LG22 and LG8). Additionally, the female map contained 3151 SNPs, and the male map contained 1220 SNPs. The lengths of the female and male maps were 2464.56 cM and 1671.97 cM, respectively. The average LG lengths of the female and male maps were 102.69 cM and 69.67 cM, respectively (Figure 1; Table 1).

### 3.3. QTL Identification and Related Genes

**Figure 2:**
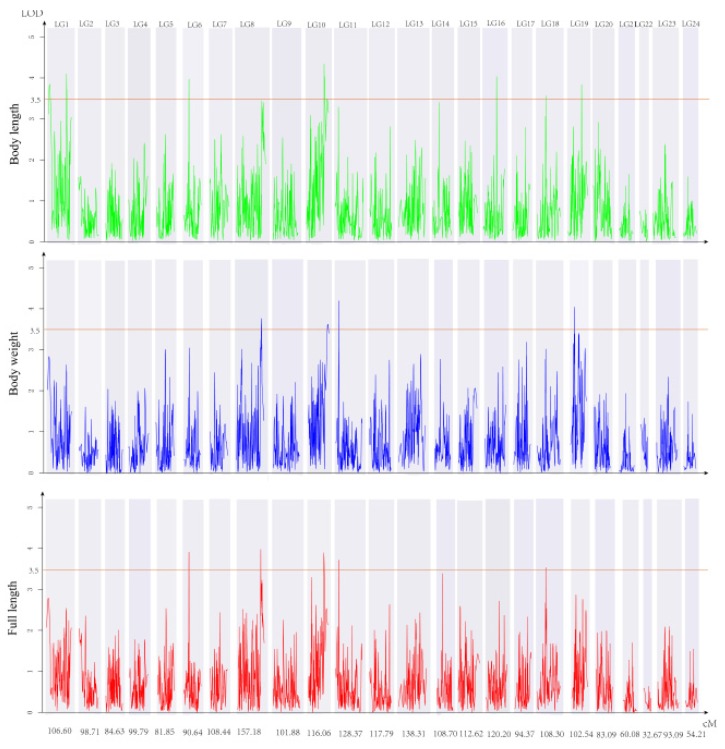
Genetic locations of growth-related QTLs on the 24 LGs of the red-spotted grouper. Green horizontal lines indicate the cutting threshold of LOD at 3.5. Green, blue, and red represent the body length, body weight, and full length traits, respectively.

In total, 17 QTLs for growth-related traits were distributed on LG1, LG6, LG8, LG10, LG11, LG16, LG18, and LG19, including 5 QTLs for BW, 5 QTLs for FL, and 7 QTLs for BL. There were 9 SNPs located on FL-related QTLs with LOD values ranging from 3.52 to 3.98, accounting for 10.7–11.9% of the phenotypic variation. Twelve SNPs were located on BL-related QTLs with LOD values ranging from 3.54 to 4.32, accounting for 10.7–12.9% of the phenotypic variation. Nine SNPs were located on BW-related QTLs with LOD values ranging from 3.55 to 4.21, accounting for 10.7–12.2% of the phenotypic variation. The LOD value of qTLG10_BL for BL was the highest value (4.32) recorded, and accounted for 12.9% of the phenotypic variation. Interestingly, markers 21190 (LG6), 449098 (LG6), 858434 (LG10), 17495 (LG10), and 343730 (qTLG18) all appeared for the BL and FL traits (Figure 2; Table 2). 

Additionally, 13 potential candidate genes were identified by the BLAST search of the orange-spotted grouper genomic region (Table 3). Among them, somatostatin (sst) was confirmed to be associated with suppressing growth hormone (GH) gene expression in the orange-spotted grouper [33], European eel (*Anguilla anguilla*) [34], goldfish (*Carassius auratus*) [35], rainbow trout [36], and grass carp (*Ctenopharyngodon idella*) [37]. The sst3 receptors on neuronal cilia suggest that they act as chemical sensors in the direct growth environment [38]. In bighead catfish (*Clarias macrocephalus*), the transcriptome data indicated that spermidine synthase (srm) was related to growth. It was demonstrated that cytochrome c (cyc1), NADH-ubiquinone oxidoreductase, and ubiquinol-cytochrome c reductase complex subunits were associated with the coordinated expression of related functional genes during muscle recovery and growth [39]. In a previous study, low-density lipoprotein receptor-related protein 1 (lrp1) affected liver lipid deposition in large yellow croaker (*Larimichthys crocea*) by influencing dietary lipid levels. Sialidase-4 (neu4) is an enzyme encoded by the *neu4* gene and was found to regulate mammal neuronal functions by degrading polySia, thereby affecting neurological function including synaptic plasticity, neurite growth, and cell migration [40]. Other genes were also found to be associated with growth-related QTLs, but their exact roles in fish have not been reported (Table 3).

### 3.4. Network Analysis Between the Candidate Genes

**Figure 3:**
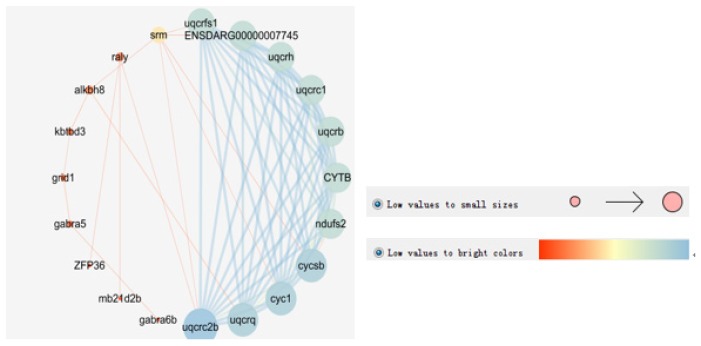
Illustration of the growth candidate gene core in Cytoscape.

In this study, 52 interacting proteins were added to provide a more comprehensive view of their interactions (Figure S1; Table S2). Fifteen proteins were found to be either directly or indirectly linked through one or more interacting proteins, suggesting that functional linkages may exist. Three biological processes were determined to be significantly involved (*p* < 0.05) based on the false discovery rate correction in this network, including mitochondrial electron transport, the ATP metabolic process, and the purine ribonucleoside monophosphate metabolic process (Table 4). One molecular function and one cellular component Gene Ontology (GO) term were enriched. Cytoscape v3.7.1 was subsequently used to visualize the STRING data (Figure 3).

## 4. Discussion

### 4.1. Molecular Marker Development and Utilization of the Genetic Map and QTL Analysis

Molecular marker technology has revolutionized research on aquaculture genetic studies. In the past, many molecular markers were used to study the genetic diversity of different aquatic organisms, including allozymes, mitochondrial DNA, restriction fragment length polymorphisms (RFLPs), random amplified polymorphic DNA (RAPD), amplified fragment length polymorphisms (AFLPs), simple sequence repeats (SSRs), microsatellite DNA, and SNP markers [53]. To date, numerous linkage maps in over 30 fish species have been constructed in aquaculture research. Meanwhile, several QTLs for essential traits were identified in more than 20 fish species between 2015 and 2019 (Table S3). Most of the published linkage maps for fish were based on SSR and SNP markers. Among them, 4 genetic maps were constructed using SSR markers, 4 were constructed through the combination of SSR and SNP markers, and 23 were developed using SNP markers. Moreover, the Nile tilapia’s linkage map contains the maximum sample capacity (689) [54]. The Atlantic salmon’s linkage map contained the largest number of markers (96,396 SNPs) in these maps [55], and the total length of the southern catfish’s map was the longest (5918.31 cM) [10]. The average locus interval between the snapper’s map was the lowest (0.129 cM) [12]. Growth traits and disease resistance are essential economic properties of fish affecting its production. Thus, QTLs related to growth and disease resistance traits have been well-studied. Although most aquatic animals exhibit high polymorphism, the linkage map generated using F_1_ (first generation) populations could meet the needs of most aquatic animals, according to previous studies [56]. Clearly, molecular markers are important tools for identifying and characterizing diverse genotypes. Additionally, genome-wide SNP discovery and linkage map analyses can lead to the discovery of thousands of markers in models or other organisms regardless of genome size or reference genome.

### 4.2. High-Density SNP Genetic Map Construction in the Red-Spotted Grouper Based on RAD-Seq

In groupers, eight genetic maps have been published previously: 5 maps were constructed using SSR markers and 3 were developed using SNP markers (Table S3). Dor et al. (2014) published a linkage map consisting of 48 white grouper individuals based on 222 microsatellite markers. The female and male maps comprised 24 LGs with 203 and 202 markers spanning 1053 and 886 cM with an average marker interval of 5.8 and 5.0 cM, respectively [17]. Liu et al. (2013) constructed linkage maps of kelp grouper using 222 SSR markers. The male map consisted of 23 LGs with 161 markers, and the female map consisted of 25 LGs with 173 markers. The total lengths of the male and female maps were 650.5 and 944.4 cM with an average marker interval of 5.0 and 6.7 cM, respectively [18]. An SSR-based map of the red-spotted grouper was previously completed by Watanabe et al. (2011) [16]. However, it is costly and time-consuming to identify candidate genes or construct a linkage map with a large number of SSR markers. As an alternative, RAD tag sequencing was developed and has been extensively used to generate SNP genotype data at the population level. The main reason for its success is that RAD-Seq does not require any previous genomic knowledge. The existing RAD methods mainly include the original RAD-Seq [10,57], IIB-restriction site-associated DNA (2b-RAD) [58,59,60], and double digest restriction site-associated DNA (ddRAD) [4,61]. The first SNP-based linkage map of the orange-spotted grouper was constructed based on RAD-Seq containing 4608 SNPs with an average marker interval of 0.56 cM. The total lengths of the female and male maps were 1370.9 and 1335.5 cM, respectively [14]. 

In this study, a linkage map of the red-spotted grouper using RAD-Seq was constructed for the first time using 3,435 RAD markers genotyped from 144 individuals. The length of the consensus map was 2300.12 cM with an average inter-location space of 0.67 cM. The total lengths of the female and male maps were 1370.9 and 1335.5 cM, respectively, indicating a substantial female bias in recombination with a female-to-male recombination ratio of 1.47:1. Sex-specific differences in recombination rates are common and have been reported in both vertebrates and invertebrates. In organisms with chromosomal sex determination, the meiotic recombination rate of males is substantially lower than females [62]. This pattern of sex-specific recombination has also been found in many fish species, including Asian seabass (*Lates calcarijer*) (the rate of total lengths of female map to male map, F:M = 1.12:1) [63], common carp (F:M= 1.23:1) [64], Atlantic salmon (F:M=1.38:1) [5], and snapper (F:M = 1.62:1) [12], as well as other aquatic animals [62,65]. There are several possible explanations for the mechanism of sex-specific recombination rates: (1) germ cells develop in different environments, with heterogametic sex and temporal differences during the beginning of meiosis between the sexes; (2) there are differences between ovocytes and sperm in homolog pairing and synapsis during meiosis [66]; and (3) at the individual chromosomal level, recombination usually varies regionally by sex with increased recombination exhibited in regions proximal to centromeres in females and telomeres in males [67]. 

### 4.3. QTL Analysis Based on the High-Density SNP Genetic Map

Mapping QTLs contributes to our understanding of the underlying genetic mechanisms of economic traits. Like most economic traits, growth is a quantitative trait regulated by multiple genes from different genomic regions. Mainly, growth traits include full length, body length, body weight, and head length. QTL mapping is a practical approach for identifying these regions. Recently, along with the successful construction of high-density genetic linkage maps, a multitude of QTLs related to growth traits have been detected in snapper [59], channel catfish (*Lctalurus punctatus*) [68], yellow drum (*Nibea albiflora*) [69], golden pompano [57], Mandarin fish (*Siniperca chuatsi)* [70], Pacific bluefin tuna (*Thunnus orientalis*) [71], pikeperch (*Sander lucioperca*) [72], blunt snout bream (*Megalobrama amblycephala*) [73], Atlantic salmon [55], large yellow croaker [74], and crucian carp [75]. No QTLs have been reported in the red-spotted grouper until now. However, several QTLs governing growth have been identified in the orange-spotted grouper, where 27 significant QTLs were detected in the genetic map, and 17 candidate genes were identified from these QTL regions. 

In this study, based on the high-density genetic linkage map of the red-spotted grouper, 17 growth trait-related QTLs with a LOD ≥3.5 were identified on eight LGs. Many QTLs exhibited pleiotropy. Namely, qTLG6-FL (21190, 449098) and qTLG6_BL (21190, 449098) were identified in a 32.24–32.59 cM interval on LG6; qTLG10-FL (858434, 17495) and qTLG10_BL (858434, 17495) were identified in the same genetic position (93.08–96.14 cM) on LG10; and qTLG11-FL (755146) and qTLG11-BW (755146) were identified in a 14.33–15.33 cM interval on LG11. The clustering of QTLs indicates that the same chromosome region is shared by various QTLs, which may be due to the high correlation between the full length and body length phenotypic traits. Identification of these pleiotropic QTLs could increase the efficiency of MAS and accelerate breeding progress. A similar result has also been reported in whiteleg shrimp (*Penaeus vannamei*) [76]. Collectively, the findings of these studies act as useful references for mining grouper QTLs. These identified candidate genes will contribute to furthering our understanding of the genetic foundation underlying grouper growth. 

### 4.4. Protein Network Analysis

Progress has been made in “omics” research (e.g., genomics, transcriptomics, proteomics, and metabolomics), which has led to vast amounts of biological data. Visualization is the easiest way to represent large biological datasets. STRING and Cytoscape software are commonly used in bioinformatic visualization studies. For example, the virulence gene prediction and construction of the protein interaction network of the orange-spotted grouper were determined using STRING and Cytoscape software, which suggested that the spleen of the orange-spotted grouper is an excellent target for studying the immune response of grouper to *Polychloride miticide* [23]. In this study, these visualization tools were used to analyze the red-spotted grouper QTL data. Twenty nodes with the greatest degree of network connection were determined using data from the Cytoscape and STRING databases. The top 20 hub genes identified were *uqcrh*, *uqcrc2b*, *uqcrc1*, *cyc1*, *cytb*, ENSDARG00000007745, *uqcrq*, *uqcrb*, *uqcrfs1*, *cycsb*, *ndufs2*, *grid1*, *gabra5*, *alkbh8*, *kbtbd3*, *srm*, *gabra6b*, *raly*, *zfp36*, and *mb21d2b*. The red-spotted grouper candidate gene with the greatest number of node networks (*n* = 11) was *cyc1*. Cytochrome c is a key protein involved in the process of energy utilization, which is the terminal step of the electron transport system. In animals, cytochrome c activity is closely related to actual oxygen consumption rates of different tissues. It is also sensitive to energy demands of exercise activity and various nutritional states [77]. Moreover, it has been demonstrated that changes in relative tissue size and cytochrome c activity could cause changes in the growth rate of largemouth bass (*Micropterus salmoides*) [78]. Therefore, cytochrome c may be an important growth target gene of the red-spotted grouper. By uncovering the protein interaction network in the red-spotted grouper, it may be possible to illuminate whole-body metabolic activity as the sum of tissue activity and physiological function.

## 5. Conclusions

A high-density genetic linkage map of the red-spotted grouper was constructed in this study. The map comprised 3435 SNPs on 24 LGs. Seventeen growth-related QTLs were identified in a genomic region. A protein–protein interaction network was also constructed. These findings provide a pivotal interactions network and will serve as a valuable resource for future investigations on the underlying molecular mechanisms of the red-spotted grouper.

## Figures and Tables

**Table 1:** Information on marker numbers and map lengths of different linkage groups (LGs) in the red-spotted grouper.

Linkage Group	Consensus		Female		Male	
	Marker	Size (cM)	Marker	Size (cM)	Marker	Size (cM)
LG1	184	106.60	146	100.12	118	126.50
LG2	75	98.71	57	70.92	75	106.19
LG3	193	84.63	193	131.33	20	40.38
LG4	145	99.79	126	95.30	74	109.50
LG5	171	81.85	157	112.50	29	44.82
LG6	140	90.64	140	99.33	13	32.17
LG7	73	108.44	70	92.97	43	73.68
LG8	251	157.18	231	160.84	40	36.32
LG9	240	101.88	213	131.38	27	44.35
LG10	174	116.06	164	118.29	65	90.05
LG11	214	128.37	200	177.05	82	79.71
LG12	155	17.79	155	130.54	63	75.75
LG13	193	138.31	164	92.35	85	119.22
LG14	80	108.70	80	95.26	80	103.62
LG15	179	112.62	169	128.81	58	74.76
LG16	174	120.20	166	118.02	75	90.29
LG17	109	94.37	90	78.62	16	35.54
LG18	118	108.30	118	100.84	29	42.07
LG19	118	102.54	118	101.20	75	87.26
LG20	114	83.09	114	94.58	66	85.05
LG21	98	60.08	98	64.59	17	69.82
LG22	20	32.67	20	45.69	7	22.78
LG23	154	93.09	105	70.01	37	60.82
LG24	63	54.21	57	54.02	26	21.32
Total	3435	2300.12	3151	2464.56	1220	1671.97
Average	143	95.84	131	102.69	51	69.67

**Table 2:** On growth-related QTLs in the red-spotted grouper. SNP: single nucleotide polymorphism; FL: full length; BL: body length; BW: body weight.

Trait	QTL	SNP ID	Genetic Position (cM)	Logarithm of Odds (LOD)	Explained Phenotype (%)
FL	qTLG6 _FL	21190	32.24_32.59	3.92	11.8
		449098			
FL	qTLG8_1	169102	137.04_137.43	3.98	11.9
		597906			
		633637			
FL	qTLG10_FL	858434	93.08_96.14	3.9	11.7
		17495			
FL	qTLG11_FL	755146	14.33_15.33	3.72	11.2
FL	qTLG18_FL	343730	41.39_42.10	3.52	10.7
BL	qTLG1_1	741153	3_9.12	3.83	11.5
		931102			
BL	qTLG1_2	110627	84.0_85.29	4.08	12.2
		365461			
BL	qTLG6_BL	21190	32.24_32.59	3.95	11.9
		449098			
BL	qTLG10_BL	858434	93.08_96.14	4.32	12.9
		17495			
BL	qTLG16	341254	75.1_75.36	4.01	12
		444247			
BL	qTLG18_BL	343730	41.39_4210	3.54	10.7
BL	qTLG19_1	978465	69.82_70.81	3.82	11.5
BW	qTLG8_2	695031	141.58_143.58	3.76	11.3
BW	qTLG10_2	819257	109.22_113.22	3.63	11
BW	qTLG10_3	689551	113.47_114.47	3.55	10.7
BW	qTLG11-BW	755146	14.33_15.33	4.21	12.6
BW	qTLG19_2	196799	23.84_24.57	4.06	12.2
		964460			

**Table 3:** Growth-related candidate genes in the red-spotted grouper.

QTL	Related Traits	SNP ID	Loci	Gene ID	Gene Description	Reference
qTLG6	FL and BL	21190	Chr4	Eco_gene_10017022	mRNA, disks large-associated protein 4-like (LOC108900233)	
qTLG6	FL and BL	449098	Chr4	Eco_gene_10020133	mRNA, somatostatin-3B-like (*somatostatin3*)	[38]
				Eco_gene_10020134	mRNA, spermidine synthase (*srm*)	[41,42]
qTLG8_1	FL	633637	Chr6	Eco_gene_10016761	mRNA, gamma-aminobutyric acid receptor subunit alpha-6 (*gabra6*)	[43]
				Eco_gene_10016762	mRNA, gamma-aminobutyric acid receptor subunit alpha-2-like (*gabra5*)	[44]
qTLG10	FL and BL	858434	Chr21	Eco_gene_10016634	mRNA, decay activator protein ZFP36-like (*zfp36*)	[45]
				Eco_gene_10016635	mRNA, leucine-rich repeat and fibronectin type III domain-containing protein 1-like protein (LOC111216932)	
qTLG10	FL and BL	17495	Chr21	Eco_gene_10021594	mRNA, leucine rich repeat and fibronectin type III domain containing 1 (*lrfn1*)	[46]
qTLG18	FL and BL	343730	Chr15	Eco_gene_10003697	mRNA, delta-like canonical Notch ligand 1 (*grid1*))	[47]
qTLG1_1	BL	741153	Chr9	Eco_gene_10012245	mRNA, RALY heterogeneous nuclear ribonucleoprotein (*raly*)	[48]
				Eco_gene_10012246	mRNA, cytochrome c1, heme protein, mitochondrial-like (*cyc1*)	[49]
qTLG1_1	BL	931102	Chr9	Eco_gene_10014886	mRNA, serine/threonine-protein kinase WNK2-like (LOC108886535)	
qTLG1_2	BL	365461	Chr9	Eco_gene_10010099	mRNA, low-density lipoprotein receptor-related protein 1-like (*lrp1*)	[50]
qTLG10_3	BW	689551	Chr21	Eco_gene_10016615	mRNA, alkB homolog 8, tRNA methyltransferase (*alkbh8*)	[51]
				Eco_gene_10016616	mRNA, kelch repeat and BTB domain containing 3 (*kbtbd3*)	
qTLG19_2	BW	96400	Chr21	Eco_gene_10000580	mRNA, sialidase-4-like (*neu4*)	[40,52]
qTLG16	BL	444247	Chr19	Eco_gene_10010609	mRNA, protein kinase C gamma type-like (LOC114557331)	

**Table 4:** Enriched GO and Kyoto Encyclopedia of Genes and Genomes (KEGG) pathway analyses in the STRING protein network.

**GO Term**	**Term ID**	**Description**	***p*-Value**
Biological process	GO:0006122	Mitochondrial electron transport, ubiquinol to cytochrome c	9.3 × 10^−4^
	GO:0046034	ATP metabolic process	5.0 × 10^−3^
	GO:0009167	Purine ribonucleoside monophosphate metabolic process	5.7 × 10^−3^
Molecular function	GO:0009055	Electron transfer activity	7.3 × 10^−4^
Cellular component	GO:0070469	Respirasome	6.2 × 10^−3^
**KEGG pathway ID**		**Description**	***p*-value**
dre00190		Oxidative phosphorylation	7.19 × 10^−14^
dre04260		Cardiac muscle contraction	2.14 × 10^−13^
dre01100		Metabolic pathways	4.09 × 10^−8^

## Supplementary Materials

The following are available online at https://www.mdpi.com/2073-4425/10/10/793/s1, Figure S1: STRING interaction network showing association between differentially expressed protein, Table S1: Phenotypic information, Table S2: The protein informations in the protein interaction networks (PPI), Table S3: Genetic maps construction for some fish species between 2015 and 2019.
